# Short-Chain Fatty Acids in Chronic Kidney Disease: Focus on Inflammation and Oxidative Stress Regulation

**DOI:** 10.3390/ijms23105354

**Published:** 2022-05-11

**Authors:** Giorgia Magliocca, Pasquale Mone, Biagio Raffaele Di Iorio, August Heidland, Stefania Marzocco

**Affiliations:** 1 Department of Pharmacy, University of Salerno, 84084 Fisciano, Italy; gmagliocca@unisa.it; 2 PhD Program in Drug Discovery & Development at University of Salerno, 84084 Fisciano, Italy; 3 Department of Medicine, Division of Cardiology, Albert Einstein College of Medicine, 1300 Morris Park Avenue, New York, NY 10461, USA; pasquale.mone@einsteinmed.edu; 4 ASL Avellino, 83100 Avellino, Italy; 5 Department of Mental and Physical Health and Preventive Medicine, University of Campania “Luigi Vanvitelli”, 80138 Naples, Italy; 6 UOC Nephrology AORN “San Giuseppe Moscati”, C.da Amoretta, 83100 Avellino, Italy; br.diiorio@gmail.com; 7 Department of Internal Medicine and KfH Kidney Center, University of Würzburg, KfH Kidney Center Würzburg, 97080 Würzburg, Germany; august.heidland@t-online.de

**Keywords:** chronic kidney disease, short-chain fatty acids, oxidative stress, inflammation, uremic toxins

## Abstract

Chronic Kidney Disease (CKD) is a debilitating disease associated with several secondary complications that increase comorbidity and mortality. In patients with CKD, there is a significant qualitative and quantitative alteration in the gut microbiota, which, consequently, also leads to reduced production of beneficial bacterial metabolites, such as short-chain fatty acids. Evidence supports the beneficial effects of short-chain fatty acids in modulating inflammation and oxidative stress, which are implicated in CKD pathogenesis and progression. Therefore, this review will provide an overview of the current knowledge, based on pre-clinical and clinical evidence, on the effect of SCFAs on CKD-associated inflammation and oxidative stress.

## 1. Introduction

The human intestinal tract hosts different microbial communities playing a pivotal role in maintaining health conditions. Gut microbiota imbalance can also exacerbate some actions promoting a cascade of metabolic abnormalities and vice versa. In numerous diseases, such as obesity, type 2 diabetes, as well as cardiovascular and auto-immune diseases, a marked alteration in microbiota composition and functions occurs [1,2]. Moreover, in Chronic Kidney Disease (CKD) patients, the gut microbiota is quantitatively and qualitatively changed with respect to healthy subjects contributing to uremic syndrome and CKD-related complications [3,4,5]. In CKD patients, microbiota metabolite changes exert major consequences. In fact, metabolites generally proven to promote health—particularly short-chain fatty acids (SCFAs)—are reduced while uremic toxins, such as indoles, ammonia, and trimethylamine N-oxide, produced by gut microbiota, accumulate—both for their overproduction and for the reduced excretion by impaired kidney function—thus enhancing CKD development and progression [6,7,8,9]. Lowered SCFAs production results in impaired CKD due to gut dysbiosis and also a decreased consumption of dietary fibre that, on the one hand, reduces SCFAs production and, on the other are, involved in increased amino nitrogen, which can be transformed into uremic toxins by gut microbiota [10]. The accumulation of these gut-derived compounds correlates with systemic inflammation and protein wasting and enhances cardiovascular complications in these patients [11]. The dysbiotic gut microbiome in CKD is associated with immune dysregulation, insulin resistance, cardiovascular disease, as well as local (gut) and systemic inflammation and oxidative stress conditions [12,13,14,15]. Thus, there has been a great interest in potentially healthy microbiota metabolites such as SCFAs, which levels result in impaired CKD. 

SCFAs are produced in the distal small intestine and the colon by anaerobic bacteria and are the end products of fermentation from complex carbohydrates that are indigestible by the human host [16]. The three major SCFAs, consisting of one to six carbon atoms, produced by gut bacteria are acetic (two carbons), propionic (three carbons), and butyric acids (four carbons). SCFAs contribute to the health of the gut (microbiome and mucosa) and the overall health of the host, with properties including anti-diabetic, anti-cancer, antibacterial, anti-inflammatory, and anti-oxidative effects [17,18]. On the other hand, lower SCFA levels contribute to different diseases such as inflammatory bowel disease [19,20], rheumatoid arthritis, and multiple sclerosis [21,22]. Moreover, SCFAs supplementation exerts anti-inflammatory actions, both at the intestinal and cardiovascular levels [23,24], and influences immune reactions [25,26,27]. Considering the anti-inflammatory potential of SCFAs, their reduced levels in CKD, and that the CKD is associated with systemic, chronic microinflammation and oxidative stress conditions that contribute to both the disease progression and to its related complications, SCFAs and SCFAs producing microorganisms could be one of the missing pieces of the puzzle in CKD-associated inflammation and oxidative stress. This review will provide an overview of the current knowledge on the effect of SCFAs on CKD-associated inflammation and oxidative stress. 

## 2. Short-Chain Fatty Acids (SCFAs)

Short-chain fatty acids (SCFA) are the end products of microbial fermentation activity. Dietary fibre escapes digestion and absorption in the small intestine and is metabolised in the colon and cecum, from which mainly acetate, propionate, butyrate, and formate are generated; while lactate is obtained from the fermentation of selected non-digestible carbohydrates, which are often also rapidly metabolised into acetate, propionate, and butyrate [28,29]. Within the intestinal microbial flora, the bacterial species most involved in SCFAs production are *Butyricicoccus* spp., *Faecalibacterium prausnitzii*, *Roseburia* spp., *Bacterioides* spp. and *Bifidobacterium*, converting dietary fibre in the gut into monosaccharides through a series of reactions mediated by specific enzymes. Acetate can be produced via acetyl–CoA or the Wood–Ljungdahl pathway; propionate is produced by the conversion of succinate to methylmalonyl-CoA via the succinate pathway or can be synthesised from acrylate with lactate as a precursor via the acrylate pathway and via the propanediol pathway; butyrate is formed either via the classical pathway by reduction by phosphotransbutyl kinase and butyrate kinase, or via the butyryl-CoA/acetate CoA-transferase pathway. In addition, some microbes in the gut can use both lactate and acetate to synthesise butyrate [30,31]. However, when fermentable fibres are scarce, microbes exploit less favourable energy sources for SCFAs production, such as amino acids from dietary or endogenous proteins, or dietary fat, also producing branched-chain fatty acids [32,33]. Although the intestinal lumen is the main site of SCFA production, their concentration varies both throughout the intestinal tract and systemically [34]. Butyrate is absorbed by the intestinal epithelium and consumed locally because it is the main energy source of colonocytes [35], while propionate and acetate cross the portal vein so that the former is metabolised in the liver [36], and the latter remains the most abundant SCFAs in the peripheral circulation, reaching organs such as the brain, pancreas, muscle, and adipose tissue, where it regulates several physiological functions [37]. In fact, SCFAs also play a key role in organs outside the digestive tract because numerous transmembrane proteins, receptors and transporters that specifically bind SCFAs and other monocarboxylic acids are expressed in a wide variety of cells [38,39]. Thus, SCFAs are associated with human health benefits for both their metabolic and/or structural properties and their signalling properties. 

### Mechanism, Role and Functions of SCFAs

SCFAs are versatile molecules involved in cell signalling in a wide range of physiological and pathological conditions [40]. Numerous pieces of evidence report that the disruption of SCFAs generated by the gut microbiota is associated with diseases, including inflammatory bowel disease, obesity, diabetes mellitus type 1 and 2, autism, major depression, colon cancer, and renal diseases [41,42]. The functions of SCFAs are mainly related to their activation of free fatty acid receptors (FFARs) belonging to the family of orphan G-protein-coupled receptors (GPCRs) and olfactory receptors (Olfr) or as histone deacetylase inhibitors (HDACs). FFARs are G-protein-coupled transmembrane receptors that bind fatty acids with carbon chains of different lengths. In particular, SCFAs are ligands of the GPR41 and GPR109A receptors coupled to the Gi/0 protein and the GPR43 receptor coupled to both Gi/0 and Gq proteins. GPR109A responds predominantly to butyrate, which also has a high affinity for the GPR43 receptor, while propionate is a potent agonist for both GPR41 and GPR43; acetate is more selective for the GPR43 receptor. Furthermore, both acetate, propionate, and butyrate also bind to the Olfr78 receptor [39,43]. In addition to their metabolic and structural roles, SCFAs possess several metabolic and signalling properties. Indeed, after intestinal microbial fermentation, they can act locally (Table 1; [44,45,46]). In the intestine, butyrate is used more as an energy source for colonocytes and can be converted into glucose by intestinal gluconeogenesis, leading to satiety and a decrease in hepatic glucose production. Butyrate binds to the GPR109A receptor, which is mainly expressed intestinally on the apical membrane of colonic and small intestinal epithelial cells, and whose activation is responsible for both the activation of the NLRP3 inflammasome, which is essential for intestinal homeostasis, and anti-inflammatory effects through increased IL (Interleukin)-18. Butyrate also binds to the GPR109A expressed on dendritic cells (DCs) and induces the differentiation of naive T cells into Th1 and Th17 cells and the increase in IL-10 in T-Reg cells, providing an anti-inflammatory function and enhancing the intestinal immune response. Moreover, in the intestine, but to a lesser extent, acetate and propionate bind to the GPR43 receptor, whose signalling functions are mediated by the Gq protein to induce the release of pancreatic peptide YY (PYY) and Glucagon-like peptide 1 (GLP-1) and influence satiety and intestinal transit. The binding of propionate and acetate to the intestinal receptor GPR43 also induces the activation of the transcription factor forkhead box P3 (FOXP3) by regulatory T lymphocytes, resulting in cell expansion and differentiation into mast cells, neutrophils, and eosinophils, respectively, implementing intestinal immunity. Then, SCFAs bind to the receptors on the immune cells of the lamina propria and enteric nervous system cells, where propionate activates the GPR41 receptor and stimulates motility and secretory activity in the colon and intestine [24,47,48,49,50]. In addition to acting locally, SCFAs from the gut can also be absorbed into the bloodstream either through anion exchange between SCFAs and HCO_3_ or through the membrane by a diffusive process promoted by the pH gradient during the diffusion of protonated SCFAs [51]. Systemically, however, acetate concentrations are higher than propionate and butyrate, and the functions of SCFAs mainly depend on the binding to the GPR43 and GPR41 receptors, inducing beneficial effects throughout the body. The GPR43 receptor has a potential role in inflammation and is most highly expressed in immune cells, adipose tissue and the subset of large renal vessels, renal afferent arteriole and juxtaglomerular apparatus, where it is involved in the regulation of renin secretion. The GPR41 receptor is expressed in the peripheral nervous system and adipose tissue and at low levels in the spleen, lymph nodes, bone marrow, peripheral blood mononuclear cells, and blood vessel endothelial cells. In the bone marrow, the activation of GPR41 induces the hematopoiesis of DCs, and in the brain, it reduces the permeability of the blood-brain barrier, increases neurogenesis, and stimulates microglia activity. In the peripheral nervous system, it induces sympathetic activation through the release of norepinephrine, leading to an increase in heart rate, energy expenditure, and satiety. However, these events are also associated with a number of hepatic events, such as increased hepatic insulin sensitivity and the activation of AMPK-dependent signalling, reduced gluconeogenesis, and reduced lipid accumulation. GPR43, GPR41, and GPR109A promote anti-lipolytic activity through increased glucose and lipid metabolism. SCFAs are also inhibitors of intracellular HDAC [48,52,53]. SCFAs enter the cell by diffusion and/or the transport mediated by sodium channel-coupled transporter protein SLC5A8 and, through HDAC inhibition, act on epigenetic modulation. In particular, butyrate and propionate as HDAC inhibitors in the intestine and colon protect against colorectal cancer and inflammation. Systemically, however, HDAC inhibition influences gene expression to exhibit anti-tumour, anti-fibrotic, and anti-inflammatory activities. In the lungs, for example, acetate reduces asthma symptoms and increases T-reg cells through HDAC9 inhibition [54,55].

## 3. Chronic Kidney Disease (CKD)

Chronic kidney disease (CKD) is a growing global problem associated with a high risk of morbidity and mortality. This condition adversely affects both human health and the expenditure of healthcare systems worldwide [56,57]. The international guidelines provided by KDIGO define CKD as an abnormality in kidney structure or function, present for >3 months, with health implications. CKD is an irreversible clinical condition associated with a definitive alteration of renal function and structure, with a slow and progressive evolution. In addition, due to the long course of CKD, one or more episodes of Acute Kidney Disease are observed, superimposed on CKD [58,59]. The loss of renal function and the progression to end-stage renal failure are evidenced by the loss of tubular cells and their replacement by collagen scars, as well as the high density of infiltrating macrophages [59,60,61]. In addition, in kidneys with CKD, the activation of the renin-angiotensin system and a reduced number of glomeruli also induce hyperfiltration and increased tubular oxygen consumption, worsening the imbalances between oxygen demand and release [62]. However, the progressive loss of renal function is linked to inflammation, the overproduction of reactive species, decreased antioxidant defences in endothelial cells (EC), the stimulation of cross-talk between EC and macrophages, and the increased expression of adhesion molecules (E-selectin, P-selectin, ICAM-1, and VCAM-1) with infiltration by monocytes and macrophages into the activated endothelium. Neutrophils are the first cells to accumulate in the renal parenchyma, further releasing reactive molecules, proteinases, elastases, myeloperoxidases, cationic peptides, cytokines, and pro-inflammatory chemokines to recruit and activate other neutrophils but also natural killer cells, monocytes, and macrophages, exacerbating renal damage through a synergistic interaction [63,64,65]. These events, which can occur in both the renal cortex and medulla, are therefore associated with a wide range of detrimental effects such as altered renal blood flow, sodium/fluid retention, inflammation, fibrotic changes, and proteinuria [66]. An event that frequently occurs with declining kidney function is the retention of toxic metabolites that are not excreted and subsequently accumulate in the systemic circulation. These metabolites are called uremic toxins, and they lead to uremic syndrome, which, in addition to the progressive loss of kidney function, is associated with symptoms such as nausea, vomiting, fatigue, anorexia, muscle cramps, itching, altered mental status and others, leading to a reduced quality of life, morbidity, and mortality [67]. Uremic toxins can be classified according to their physico-chemical characteristics, such as water-soluble free solutes with a low molecular weight (<500 Da; e.g., Guanidine, Creatinine, Urea, Trimethylamine N-oxide, Inorganic phosphorus), protein-bound uremic toxins (<200 Da; e.g., Indoxyl Sulfate (IS), p-Cresyl Sulfate (pCS), Indole-3-acetic acid, Phenol Quinolinic acid, Putrescine), and medium molecules (≥500 Da; e.g., β2- microglobulin, Leptin, IL-6, β-trace protein, Parathyroid hormone). Despite the fact that most of these metabolites are eliminated by dialysis, except the plasma protein-bound [68], the resulting accumulation of uremic toxins is associated with CKD progression and related complications, such as cardiovascular, central nervous system, gastrointestinal, and other areas, also trigger inflammation and oxidative stress and impaired immune response [69,70,71,72,73,74,75,76,77,78].

### Gut Microbiota and SCFAs in CKD

The complex functions of the gut microbiota are related to other organs and result in the formation of an ‘-axis’ between them [79]. The gut microbiota plays an important role in kidney homeostasis, regulating the gut-kidney axis [80], and intestinal dysbiosis is implicated in the pathogenesis of various renal disorders, including urinary tract infections (UTIs) that are also related to the “intestinal bloom of uropathogens” with a prevalence of the uropathogenic *Escherichia coli* [81,82]. These infections can also evolve into pyelonephritis as a complication of an ascending urinary tract infection that spreads from the bladder to the kidneys and their collecting systems, which still results in the significant morbidity and mortality associated with the severe cases of this disease [83]. A marked gut dysbiosis is also commonly observed in CKD patients and results from qualitative and quantitative changes in the composition and metabolic activities of the gut microbiota [84]. This may be due to both the use of antibiotics and drugs (e.g., iron-containing or resin-based phosphate binders) and changes in diet, including a decrease in resistant starch and/or fibre content or restriction of fruits and vegetables, as well as a decrease in colonic transit time in patients with uremia [85,86]. Furthermore, during CKD, the colon becomes the main route for uric acid and oxalate secretion. The influx of urea, uric acid, and oxalate into the colon affects the composition and metabolism of the gut microbiota, promoting the overgrowth of urease-producing bacteria and changes in the growth of the bacterial communities themselves [23]. Thus, an increase in Phyla *Actinobacteria*, *Firmicutes* and *Proteobacteria* microbes and a decrease in *Bifidobacteria* and *Lactobacilli* and SCFA levels have been reported in the course of CKD and in patients with end-stage renal disease [4,87]. These aspects reflect the evidence that SCFA levels progressively decrease during the different stages of CKD and, ultimately, in dialysis patients [88]. SCFAs produced by bacteria in the kidney protect tubular cells from oxidative stress and mitochondria biogenesis, reduce renal ischaemia-reperfusion injury, inflammation, reactive molecules, and immune and apoptotic cell infiltration in damaged kidneys [89]. Thus, the dysbiotic microbiota produces both a large amount of NH_3_/NH_4_OH that influences the pH of the intestinal lumen and toxic metabolites such as indoles and phenols that are further metabolised in the liver and intestine into pCS, IS, and TMO. Generally, pCS accumulates in tubular cells and binds to OAT receptors located on the basolateral membrane of renal proximal tubular cells and generates reactive oxygen species, whereas IS binds to OAT receptors and activates NF-κB and AP-1-dependent gene transcription, inducing inflammation and nephrotoxicity [90]. Thus, these toxic metabolites may lead to accelerated renal damage by both promoting the progression of oxidative stress and inflammation and by promoting the alteration of the gut microbiota, which, by further producing gut-derived toxins, also alters the function of the intestinal epithelial barrier. At the same time, these toxic metabolites are absorbed through the damaged intestinal barrier and released into the systemic circulation. Indeed, there is considerable evidence to suggest that gut dysbiosis may contribute to the progression of some of the events that occur over the course of CKD, such as oxidative stress, endotoxemia, inflammation, and an increased prevalence of comorbidities [91,92,93,94]. Thus, it seems clear that there is a close relationship between the gut microbiota and renal function that is implicated in renal physiology and disease conditions.

## 4. Inflammation and Oxidative Stress in CKD

Chronic inflammation is a common comorbid condition in CKD. The increased production and reduced clearance of pro-inflammatory cytokines, oxidative stress, and acidosis contribute to the chronic inflammatory state but also to metabolic alterations and chronic and recurrent infections, especially in dialysis patients. Furthermore, metabolic alterations and intestinal dysbiosis create additional inflammatory stimulation with the involvement of the cells of the innate immune response system [95,96,97]. Among the inflammatory markers in CKD, IL-1, IL-6, TNF (Tumor Necrosis Factor)-α, C-reactive protein (CRP), adipokines, adhesion molecules, and the CD40 ligand are particularly important and associated with many complications (e.g., malnutrition, coronary calcification, atherosclerosis, atrial fibrillation, left ventricular hypertrophy, heart failure, insulin resistance, oxidative stress, endothelial dysfunction, mineral and bone diseases, anaemia, and erythropoietin resistance). In addition to being produced by lymphocytes, these pro-inflammatory factors are produced by visceral adipose tissue, which becomes dysfunctional during CKD by expressing a high level of pro-inflammatory cytokine mRNA [98]. Alongside the levels of these cytokines, there is also an increase in pro-inflammatory enzymes such as cyclooxygenase-2 (COX-2) and inducible nitric oxide synthase (iNOS), which are positively regulated by the activation of NF-κB in CKD [99,100]. Several studies have also demonstrated that uremic toxins, such as IS, are able to increase the levels of TNF-α and IL-6, causing an exacerbation of the inflammatory state and favouring oxidative stress [101,102]. Indeed, oxidative stress is also frequently observed in the early stages of chronic renal failure onwards and, in addition to being a non-traditional risk factor for all causes of mortality, tends to exacerbate during the course of the disease and can sometimes persist to a certain degree after kidney transplant [103,104]. Oxidative stress is responsible for several pathological conditions that are considered risk factors for CKD, such as diabetes, hypertension, and atherosclerosis, and is also responsible for the progression of kidney damage, which leads to renal ischemia, glomerular damage, cell death, and apoptosis, which also exacerbate the severe inflammatory processes already underway [105,106]. Oxidative stress is a condition of imbalance between the excessive production of oxidants and the reduced capacity of antioxidant systems, which leads to metabolic dysregulation and the oxidation of lipids, DNA, and proteins, as well as affecting the cellular activity and inhibiting the activity of cytoprotective enzymes [102,107]. Oxidative stress is linked to the production of highly reactive intermediates, reactive oxygen (ROS), and nitrogen (RNS) species, whose excessive generation is associated with impaired electron transport chains, reduced ATP synthesis, mitochondrial dysfunction, cell damage, apoptosis, and even damage to all of the cellular constituents [108]. Mitochondria are mainly responsible for the production of reactive molecules through the electron transport chain, especially ROS, which are also able to improve the inflammatory response. Indeed, during the pathogenesis of kidney disease, mitochondria in damaged cells become one of the main sources of excess ROS, which in turn implements the activation of transcription factors NF-κB, AP-1, and p53, exacerbating the production of pro-inflammatory cytokines and chemokines such as IL-1β, IL-6, IL-8, IL-1, and TNF-α, monocyte chemoattractant protein-1 (MCP-1), interferon-invasive protein-10 (IP-10), molecules of adhesion such as selectin, ICAM, VCAM, ELAM, inflammatory enzymes, such as iNOS and COX-2, and further ROS/RNS [109,110,111,112,113]. The main markers of oxidative stress that have significantly elevated levels in the blood and/or circulating tissues in patients with CKD are malondialdehyde (MDA), a low-density oxidized lipoprotein, advanced glycation end products, and l’8-hydroxide-oxyguanosine. For example, the interaction between AGE and the RAGE receptor induces the activation of the MAP kinase transduction pathway, leading to the nuclear translocation of NF-κB and the activation of second messengers, resulting in an increase in cytokines, pro-inflammatory enzymes, and adhesion molecules [114,115,116]. Thus, as oxidative stress can further exacerbate inflammation, inflammation and oxidative stress are important mediators in the development and progression of kidney disease and associated complications, where one generates and amplifies the other, and the antioxidant systems are severely compromised [94]. In fact, this condition also depends on the reduced activation of antioxidant responses, such as the transcription factor Nrf2 (Erythroid-related nuclear factor 2), the main cellular defence factor that regulates the genes coding for antioxidant and detoxifying proteins and enzymes. Generally, Nrf2 is in a quiescent state sequestered by the cytosolic repressor Keap1 (Kelch-like ECH-associated protein 1), which also promotes its rapid proteasomal degradation; in contrast, under oxidative and electrophilic stress conditions, Nrf2 is released by Keap1, which in this case acts as an electrophilic sensor and, together with the small Maf protein (sMAF), binds to the antioxidant response element (ARE) in the promoter region of genes coding for phase II and antioxidant enzymes to counteract oxidative stress [117,118,119]. In addition, Nrf2 also directly suppresses the expression of pro-inflammatory NF-κB target genes by binding to their promoters and inhibiting their transcription [120]. However, in the course of CKD, the excessive production of ROS reduces the activation of Nrf2, and its deficiency increases the susceptibility to kidney damage. Indeed, several studies have shown that during CKD, Nrf2 has a renoprotective effect by controlling uremic inflammation and improving antioxidant defences, leading to a reduction in renal fibrosis, tubular damage, and renal hypoxia [121].

### SCFAs, Inflammation and Oxidative Stress

**Figure 1:**
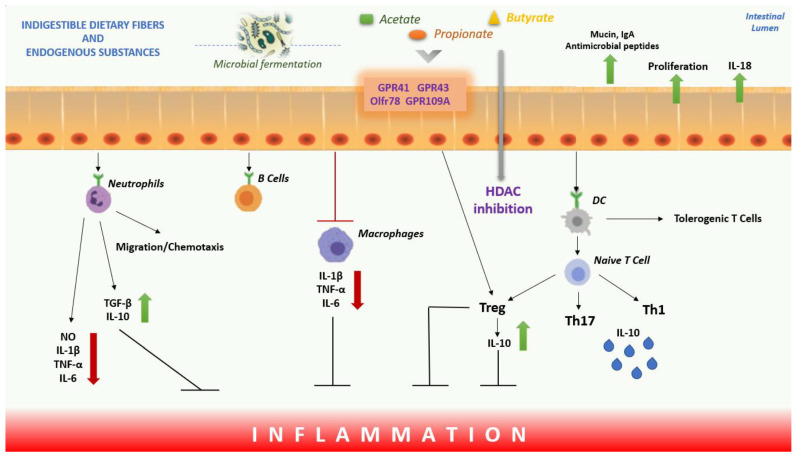
Overview of the effect of the SCFAs on inflammation. In the intestinal lumen, SCFAs induce the secretion of IL-18, MUC2 and antimicrobial peptides from intestinal epithelial cells, induce IgA secretion from B lymphocytes and regulate tight junction expression. SCFAs bind to GPR41, GPR43, GPR109A receptors and activate Olfr78 receptor signalling to regulate T cell function increasing the generation of Th1 and Th17 cells and promoting the production of T cells that release IL-10 and T regs. SCFAs act on DCs to limit the expression of T cells activating molecules, resulting in the generation of tolerogenic T cells rather than inflammatory T-cells. SCFAs also reduce neutrophil recruitment, with increased levels of TGF-β, IL-10 and decreased levels of IL-6, IL-1β, NO, and TNF-α. Instead, through HDAC inhibition, they influence the inhibition of nuclear factor NF-κB, to inhibit inflammation. Abbreviations: DCs, Dendritic Cells; SCFAs, Short-chain Fatty Acids; GPCRs, G-Protein-coupled Receptors; HDAC, Histone Decetylase; NO, Nitric Oxide; TGF-β, Transforming Growth Factor beta; TNF-α, Tumor Necrosis Factor alpha; IL, Interleukin; Mucin, MUC2; NF-κB, Nuclear Factor Kappa-light-chain-enhancer of Activated B cells.

SCFAs produced by the intestinal microbiome are able to act on inflammation and oxidative stress through complex mechanisms of regulation, and, moreover, they also regulate the immune response. SCFAs suppress inflammation in many organs by reducing the migration and proliferation of immune cells and cytokine levels and by inducing apoptosis [122]. Through the inhibition of HDAC, they influence the inhibition of the nuclear factor, NF-κB, and the transcription of genes that code for pro-inflammatory cytokines. Furthermore, they are also able to reduce the inflammatory response through the reduction in neutrophil recruitment, with increased levels of TGF-β and IL-10 and reduced levels of IL-6, IL-1β, NO, and TNF-α. At the same time, SCFAs promote the production of T cells that release IL-10 and T-reg to prevent inflammatory responses and act on DCs to limit the expression of T cell activating molecules, resulting in the generation of tolerogenic rather than inflammatory T cells, thus reducing inflammatory responses. SCFAs can also modulate the immune response due to a direct effect on T cells, binding to GPR41, GPR43, and GPR109A receptors and activating Olfr78 receptor signalling to regulate T lymphocyte function by increasing the generation of Th1 and Th17 cells to improve immunity (Figure 1). Butyrate, for example, has shown both an inhibitory effect on the formation of NLRP3 inflammasomes and an improvement in tight junction function in intestinal and vascular endothelial cells [123,124]. Moreover, butyrate, through HDAC inhibition, was able to modulate the immune response by reducing iNOS levels and NF-κB activation [125].

**Figure 2:**
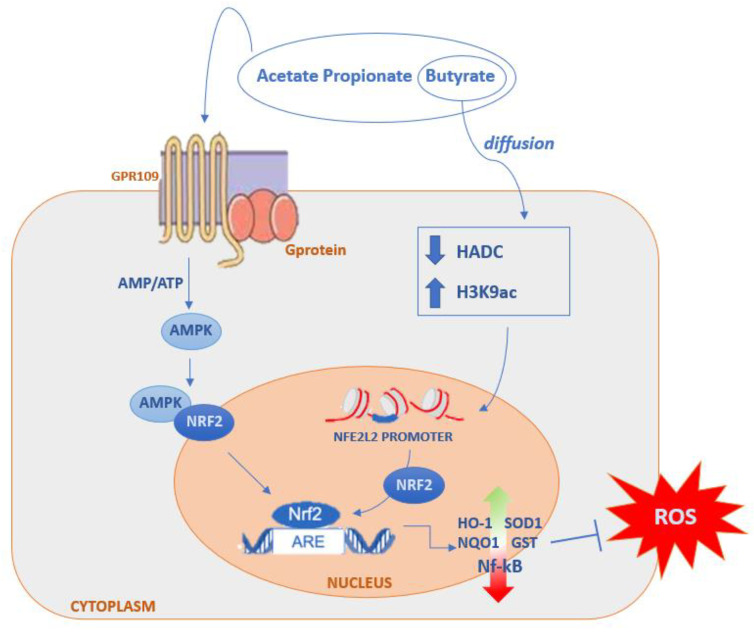
Direct and indirect mechanism of SCFAs on Nrf2 activation for modulation of oxidative stress. Binding of SCFAs to GPRC receptors induces direct activation of the nuclear factor Nrf2. Butyrate, on the other hand, also has a synergistic effect on Nrf2 activation because it diffuses into the cell lumen and, through HDAC inhibition, increases the production of histone H3K9ac, thus inducing an epigenetic modification on the Nrf2 promoter, indirectly activating Nrf2-dependent gene translocation and transcription. Abbreviations: AMPK, Activated Protein Kinase; HDAC, Histone Deacetylase; Nrf2, Nuclear Erythroid-Related Factor 2; ARE, Antioxidant Response element; HO-1, Heme Oxygenase-1; NQO1, NAD(P)H Quinone Dehydrogenase-1; NF-κB, Nuclear Factor Kappa-light-chain-enhancer of Activated B cells; SOD1, Superoxide Dismutase 1; GST, Glutathione S-transferase.

Furthermore, numerous studies have shown that SCFAs, particularly butyrate and propionate, were also able to modulate the Keap1-Nrf2-dependent cellular signalling pathway to maintain redox homeostasis through both direct and indirect mechanisms (Figure 2; [125,126,127,128,129,130]). Butyrate, through the recognition of the GPR109A receptor, induces the activation of the nuclear factor Nrf2, which encodes antioxidant enzymes for the inactivation of ROS [108]. Furthermore, butyrate has a synergistic action on the activation of Nrf2 because, by spreading in the cell lumen, it inactivates HDAC and consequently increases the production of histone H3K9ac thus inducing an epigenetic modification on the Nrf2 promoter, as demonstrated through various studies [125,126,131,132,133]. Acetate, propionate, and butyrate can synergistically activate the translocation of Nrf2 through the recognition of GPR41 and GPR43 receptors [134,135,136].

## 5. Effects of SCFAs in CKD

CKD is linked to inflammation, oxidative stress, and dysbiosis of the immune system. These factors contribute to the progressive deterioration of renal function, loss of blood pressure control, metabolic dysfunction, and a loss of functional integrity of the intestinal epithelial barrier. Furthermore, the perpetuation of systemic inflammation and oxidative stress, together with the accumulation of toxic metabolites, are responsible for the onset of all comorbidities associated with CKD, such as changes in the cardiovascular, pulmonary, ocular, central nervous, musculoskeletal, gastrointestinal, mitochondrial, and immune systems [73,137,138,139,140,141,142]. However, in recent years, the gut microbiota has been assumed to play a central role in renal disease through the production of SCFAs, which have been shown to ameliorate renal damage by modulating inflammatory and immune responses [140,141,142]. Numerous studies, both pre-clinical and clinical, have already demonstrated the potential beneficial effect that SCFAs could have in the course of CKD, even improving some of the secondary complications that occur.

### 5.1. Pre-Clinical Observations

In vitro studies in cellular models were used to assess the relationship between SCFAs and inflammation and oxidative stress. Huang et al. evaluated the effect of SCFAs on oxidative stress and inflammation induced by high levels of glucose and lipopolysaccharide (LPS) in mouse glomerular mesangial cells (CMG) (SV-40 MES 13) in the presence of acetate and butyrate or GPR43 agonist. The results indicated that both the treatment with SCFA and the treatment with the GPR43 agonist reduced MCP-1, IL-1β, and ICAM-1 levels. Moreover, both acetate and butyrate and the agonist GPR43 inhibited the generation of ROS and MDA and reversed the decrease in SOD induced by high levels of glucose and LPS. These pieces of evidence support the hypothesis that both SCFAs and the GPR43 signalling pathway may act as potential therapeutic targets in inflammation and oxidative stress in glomerular mesangial cells [143]. Andrade-Olivera and colleagues also confirmed that SCFAs modulated the inflammatory process. In renal tubular epithelial cells (TECs) stimulated with an inflammatory cocktail (LPS, zymosan, and TNF-α) and treated with butyrate, propionate, and acetate indicated that SCFAs reduce NF-κB activation, nitric oxide production, and ROS production in TECs. Furthermore, the translocation of hypoxia-inducible factor (HIF)-1 α transcription factor to the nucleus, a hallmark of hypoxia, was also reduced due to the role of SCFA. Therefore, treatment with SCFAs seems to counteract the inflammatory response and hypoxia in renal tubular epithelial cells. SCFAs could also modulate the inflammatory response, regulating immune cells and reducing the expression of the costimulatory molecules, CD80 and CD40, in bone marrow dendritic cells (DCs), and reducing CD8+ and CD4+ cell proliferation after treating antigen-presenting cells (APCs) from RAGKO mice with LPS, with or without SCFAs, for 24 h. Other studies performed in animal models of renal disease evaluated the effects of SCFA. In particular, acetate showed to have beneficial effects in preserving the structure of the kidney, reducing ROS, cytokines, and chemokines. Then, low mRNA levels of toll-like receptor 4, and its endogenous ligand, lower the activation of the NF-κB pathway, wherein low levels of activated neutrophils and macrophages, a low frequency of infiltrating macrophages, and a low frequency of activated DCs were observed. Acetate also increased the expression of GPR43 by modulating the expression of genes encoding for enzymes involved in epigenetic modifications and inhibited the activity of HDACs [144]. Butyrate appears to modulate the inflammatory response in vitro, also modifying the profibrotic cytokine transforming growth factor beta (TGF- β1) generation on immortalised human renal proximal tubular epithelial cells (HK-2 cells). There is strong evidence that this cytokine is involved in renal fibrosis in all renal diseases, and butyrate reduces the basal generation of TGF-β1 in renal tubular epithelial cells; in addition, butyrate mediates its effect through the inhibition of ERK/MAP kinase. This evidence was useful in confirming the role of butyrate in preventing renal fibrosis through the reduction of TGF-β1 and provided a useful basis for subsequent studies on dietary supplementation with Acacia(sen) SUPERGUM™ (gum arabic) that, increasing systemic levels of butyrate, may therefore have a potential beneficial effect in renal disease through the suppression of TGF-β1 activity [145,146,147,148]. SCFAs are, therefore, able to directly modulate some of the pro-inflammatory and oxidative stress parameters, as also demonstrated by other studies [149,150].

The effect of SCFAs in modulating inflammation and oxidative stress response was also reported in in vivo studies in animal models of chronic renal failure, which also correlated with a number of secondary complications.

Acute Kidney Injury (AKI) is an important risk factor for CKD. Therefore, Liu et al. used a mouse model of folic acid nephropathy to examine the effect of dietary fibre, from which SCFAs are derived after microbial fermentation, on the development of AKI and, consequently, on the progression of CKD. Wild-type and knockout mice for GPR41, GPR43, or GPR109A receptors in which folic acid nephropathy had been induced were fed fibre-rich diets or treated with SCFAs. The gut microbiota was examined by RNA sequencing, and an increase in *Bifidobacterium* and *Prevotella* was observed, which also increased the concentration of SCFAs in both faeces and serum. After 28 days, the animals showed improved kidney function, fewer tubular lesions, and fewer interstitial fibrosis; chronic inflammation was evaluated by the gene expression analysis of various inflammatory parameters, such as TLR-2, TLR-4, pro-inflammatory cytokines (e.g., TNF-α, IL-6, IL-18, IL-1β, IL-4, IL-10, and IFNγ), and anti-inflammatory cytokine IL-10, the activation of NLRP3 inflammasome, chemokines (e.g., CXCL2, CCL2, and CXCL10), TGF-β1 expression and pro-inflammatory enzymes (e.g., iNOS). The SCFAs treatment led to similar protection through the inhibition of HDAC and GPR41-, GPR43-, and GPR109A-dependent signalling. Thus, both dietary manipulation and SCFAs have been shown to significantly reduce the damage of AKI and, thus, the risk of CKD progression [151]. Diabetic nephropathy is a chronic inflammatory condition that often overlaps with CKD, in the pathogenesis of which oxidative stress and NF-κB signalling are mainly observed. Huang et al. evaluated the role of acetate, propionate, and butyrate both in vitro on GMC cells (SV-40 MES 13) and in different animal models such as mice with type 2 diabetes (T2D) induced by streptozotocin (STZ), diabetic nephropathy (DN), and GMC cells of high-glucose mice, but also in a high-fat diet (HFD). In GMCs, SCFAs inhibited oxidative stress by reducing ROS and MDA and increased SOD, reduced NF-κB activation, enhanced the interaction between β-arrestin-2 and I-κBα, and reduced the release of MCP-1 and IL-1 β. For in vivo studies, however, the kidneys were used for the pathology assessment, and biochemical analyses were performed. The results showed that SCFAs, particularly butyrate, improved hyperglycaemia and insulin resistance, reduced proteinuria, serum creatinine, urea nitrogen and cystatin C, inhibited mesangial matrix accumulation and renal fibrosis, and blocked NF-κB activation in mice by GPR43-mediated signalling. SCFAs ameliorated the renal damage of DT2 and demonstrated antioxidant and anti-inflammatory effects mediated by the overexpression of GPR43 [152]. These results were also confirmed by another study. Diabetes was induced by STZ in wild-type C57BL/6 and GPR43 or GPR109A knockout mice, and then they were fed fibre-rich diets followed by sodium acetate, sodium propionate, and sodium butyrate. After 12 weeks, stool, urine, and plasma samples were collected and examined. The results indicated that diabetic mice fed a high-fibre diet had less albuminuria, glomerular hypertrophy, podocyte lesions, and fibrosis and were less likely to develop diabetic nephropathy and, consequently, CKD. The fibre also promoted the expansion of SCFA-producing bacteria such as *Prevotella* and *Bifidobacterium*, which increased the faecal and systemic SCFA concentrations, and reduced the expression of genes encoding for inflammatory cytokines, chemokines, and fibrosis-promoting proteins in diabetic kidneys. In vitro studies used TEC cells and podocytes isolated from C57BL/6 mice, both treated with either acetate, propionate, or butyrate. The results indicated that SCFAs modulated inflammation by reducing the chemokines CCL2 and CXCL10 and the cytokines IL-6 and TNF-α. In addition, the expression of the fibrosis-related genes TGF-β and fibronectin was also reduced [153]. These effects depended on the modulation of inflammation by SCFAs through the GPR41 and GPR43 receptors, as also shown in other studies. Indeed, butyrate, through the activation of the GPR109A receptor in renal podocytes, influences the gene transcription of pro-inflammatory cytokines and controls inflammatory responses. This GPR109A receptor phenotype in renal podocytes was associated with an increase in podocyte-related proteins and a normalised pattern of acetylation and methylation at the promoter sites of genes that are essential for podocyte function. Thus, the protective effect of butyrate-dependent GPR109A signalling ameliorated proteinuria by preserving the podocytes on the glomerular basement membrane and attenuating glomerulosclerosis and tissue inflammation [154,155]. There is a large body of evidence that CKD is associated with impaired function and decreased integrity of the intestinal epithelial barrier. Indeed, chronic low-grade inflammation and marked alteration of the intestinal microbiota can be observed in the intestine, which, by further producing toxic metabolites, promotes increased inflammation and its progression to the systemic level. Hung et Suzuki conducted a study in which they evaluated whether fermentable dietary fibre (DF), such as unmodified guar gum (GG) and partially hydrolysed GG (PHGG), could cause an increase in SCFA concentrations and, consequently, restore intestinal barrier permeability and function, thereby also improving inflammation in cases of CKD. Thirty-three seven-week-old male mice were fed a diet supplemented with adenine for 14 days to induce CKD and were subsequently examined. Twenty-seven of these mice were then divided into three groups (CKD, CKD+GG, and CKD+PHGG), while six mice received a control diet. Pro-inflammatory parameters, such as TNF-α and IL-1β, tight junction proteins, such as zonula occludens (ZO)-1, ZO-2 and occludin, serum urea and creatinine, intestinal barrier permeability, SCFA levels, and bacterial populations were examined. The results indicated that in the mice fed with GG and PGHH, not only was inflammation reduced, but high caecal levels of SCFAs, intestinal barrier function, and bacterial population composition, in particular *Lactobacillus*, were also improved. Thus, SCFAs, produced through the intestinal fermentation of PHGG and GG and transported into the circulatory system, have been shown to suppress inflammation and renal fibrosis directly [156]. In another study, the effects of the prebiotic fibre, xylooligosaccharide (XOS), on renal function and gut microbiota in mice with adenine-induced CKD were evaluated. The mice were fed adenine for 3 weeks to induce CKD and then fed XOS for a further 3 weeks. The results indicated that XOS reduced the renal damage in CKD mice, improved intestinal bacterial populations, increased the caecal production of SCFAs and reduced the levels of the uremic toxin IS [157].

The study sections and results are summarised in Table 2.

### 5.2. Clinical Observations

Thus, it is now clear that SCFAs may have a central and promising role in the treatment of renal failure. Indeed, several clinical trials have already been initiated. In the inflammation and oxidative stress observed in CKD, uremic toxins of intestinal origin also play a central role, promoting excess morbidity and mortality. This may be due to intestinal dysbiosis and the insufficient consumption of fermentable, complex carbohydrates, which consequently lead to reduced SCFA concentrations. Thus, a pilot study was conducted at the ‘A Landolfi’ Hospital (Solofra, Italy) in which 20 stable patients aged between 18 and 90 years were recruited, of which the most frequent causes of renal failure were diabetes mellitus and chronic glomerulonephritis. All of the subjects suffered from vascular and cardiac complications. Biochemical analyses were performed on the sera, and elevated levels of inflammatory and pro-oxidant markers were observed. However, when SCFAs, in particular sodium propionate, were administered, significant improvements were observed: no patient discontinued the treatment, and their body weight remained stable, there was a significant decrease in pro-inflammatory and pro-oxidant parameters such as high-sensitivity C-reactive protein (hs-CRP), IL-2, IL-6, IL-10, IL-17a, TNF-α, INF (Interferon)-γ, TGF-β, and endotoxins/lipopolysaccharides compared to the significant increase in the anti-inflammatory cytokine IL-10. In addition, the levels of MDA and uremic toxins, indoxyl sulphate and p-cresyl sulphate, were reduced. This study, therefore, from its conclusion, provided new information on the benefits of SCFAs for treating systemic inflammation, oxidative stress, and metabolic disorders [158]. Considering that elevated blood pressure and cardiovascular morbidity occur very often in patients with CKD. Some studies have shown that SCFAs can improve cardiovascular outcomes in CKD patients with kidney disease [159,160]. In fact, in 2019, the first clinical study was conducted to verify that SCFAs can bind to both the Olfr78 receptors expressed in the kidney on the afferent arteriole of the juxtaglomerular apparatus involved in the production of renin and the GPR41 receptor in the renal vascular system with contrasting effects on blood pressure [161,162,163,164]. Jadoon and colleagues examined the potential link between SCFAs and cardiovascular outcomes in patients with chronic renal failure. In a subcohort of 214 patients with CKD in the Clinical Phenotyping Resource and Biobank Core (CPROBE), including 81 patients with coronary artery disease (CAD) and self-reported cardiovascular disease (CVD), they measured the plasma levels of SCFAs by liquid chromatography-mass spectrometry and high-performance liquid chromatography. The results showed improved cardiovascular function in CKD patients, which was linked to significantly higher levels of SCFAs [165]. Furthermore, SCFAs have also been shown to significantly decrease systolic blood pressure in hemodialysis patients [166]. SCFA levels improved by diet also seems to have a positive effect on CKD. In fact, diet can improve the course of CKD by reducing urea levels [167], metabolic acidosis [168], and insulin resistance [169], as well as positively modulating the intestinal microbiome and, consequently, increasing SCFA concentrations [170,171]. Type 2-resistant starch-enriched biscuits (RS2) were administered to hemodialysis patients with chronic renal failure for 4 weeks. The results showed an increase in SCFA-producing bacteria *Roseburia* and *Ruminococcus gauvreauii* and a downregulation of the pro-inflammatory parameters [172]. While, in the course of another prospective, randomised, crossover study (Medika Study), for the first time, the effect of different diets on the modulation of the intestinal microbiota and, consequently, on the modification of the serum levels of IS and pCS were evaluated in patients with chronic renal failure. Sixty patients with grade 3B-4 chronic renal failure were recruited and given a free diet (FD), a very-low protein diet (VLPD) and a Mediterranean diet (MD). The stool and serum samples were collected at the end of each dietary regimen for the evaluation of IS and pCS levels or serum D-lactate levels. The results indicated that MD and VLPD increased bacterial species with anti-inflammatory potential and butyrate producers, circulating levels of IS and pCS were reduced, and an improvement in structural integrity and intestinal permeability was observed. Furthermore, VLPD reduced serum D-lactate and improved systolic blood pressure [173]. Wu and colleagues, on the other hand, evaluated changes in the composition of the gut microbiota in patients with chronic renal failure who followed a low-protein diet (LPD). In this study, 43 patients with chronic renal failure were involved, and changes in bacterial population, SCFAs production and uremic toxins were evaluated. These results also confirmed that nutritional therapy based on low protein intake improved renal function, reduced IS and pCS levels, and increased butyrate-producing bacterial populations [174].

The study sections and results are summarised in Table 3.

## 6. Conclusions

**Figure 3:**
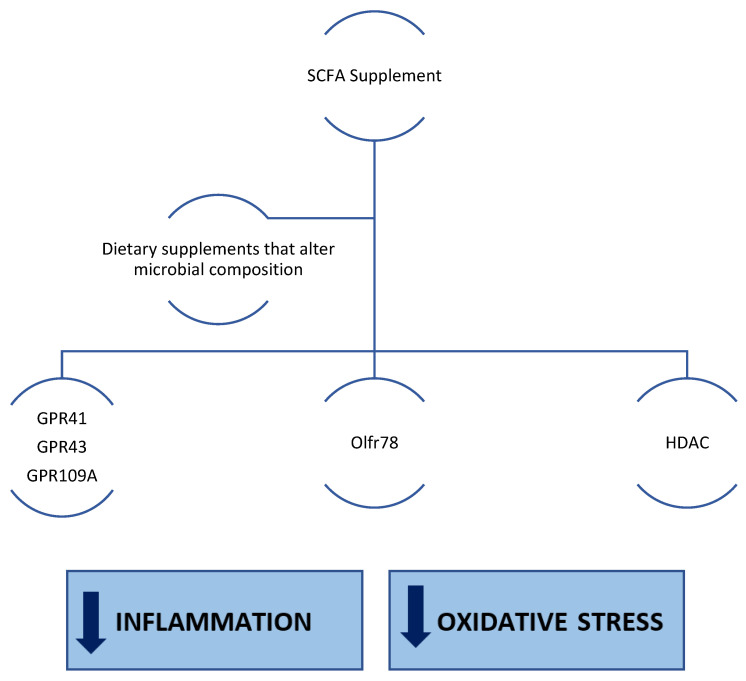
SCFAs result in increased activity on G-protein-coupled receptors and enhanced epigenetic regulatory activity through HDAC. SCFAs could be able to act positively on pro-inflammatory pathways (e.g., by negatively modulating the NF-κB signalling pathway) and pro-oxidant pathways (e.g., by positively modulating the Nrf2 pathway).

SCFAs produced by microbial fermentation have been widely shown to reduce inflammation and oxidative stress, which are characteristic of several chronic diseases. CKD is a serious health problem, not least because of the systemic complications associated with it, and it is also a chronic condition that is very difficult to manage because of the underlying disease mechanisms. Therefore, the supplementation of short-chain fatty acids (e.g., acetate, propionate, or butyrate), either directly or by modulating the gut microbiota in favour of SCFA-producing bacterial species, including through dietary fibre or nutritional therapies, could have a positive impact on the management of chronic renal failure (Figure 3). However, further studies are still needed.

## Figures and Tables

**Table 1:** Acetate, propionate, and butyrate are formed in the human colon in an estimated ratio of approximately 3:1:1. Different bacteria are involved in SCFAs production, and once produced, SCFAs are able to bind to different receptors. In the table are indicated the receptors for which each SCFA has a major affinity and their intestinal and non-intestinal expression.

SCFAs	Producers	Binding	*Intestinal Expression*	*Non Intestinal Expression*
**Acetate**	*Akkermansia muciniphila*, *Bacterioides* spp., *Bifidobacterium* spp., *Prevotella* spp., *Ruminococcus* spp., *Blautia hydrogenotrophica*, *Clostridium* spp., *Streptococcus* spp.	**GPR43**	Colonic, Small intestinal epithelium, Colonic lamina propria cells, Leukocytes in small intestinal	Polymorphonuclear cells, Adipocytes, Skeletal muscle, Heart, Spleen
		**Olfr78**		
**Propionate**	*Bacterioides* spp., *Phascolarctobacterium succinatutens*, *Dialister* spp., *Veilonella* spp., *Megasphera elsdenii*, *Coprococcus catus*, *Ruminococcus obeum*, *Salmonella* spp., *Roseburia inulinivorans*	**GPR43**	Colonic, Small intestinal epithelium, Colonic lamina propria cells (mast cells), Pancreas, Gut enteroendocrine cells located in the crypts and lower part of the villi	Spleen, Bone marrow, Lymph nodes, Adipose tissue, Periportal afferent system, Peripheral nervous system, Peripheral blood mononuclear cells
		**GPR41**		
		**Olfr78**		
**Butyrate**	*Coprococcus viene*, *Coprococcus eutacus*, *Anaerostipes* spp., *Coprococcus catus*, *Eubacterium rectale*, *Eubacterium hallii*, *Faecalibacterium prausnitzii*, *Roseburia* spp.	**GPR109A**	Apical membrane of colonic/small intestinal epithelium, Macrophages, Monocytes, Neutrophils, Dendritic cells	Adipocytes (white and brown), Epidermal Langerhans cells, Retinal pigment epithelium
		**GPR41**		
		**GPR43**		

**Table 2:** Pre-clinical studies report a related improvement in SCFA levels both in in vitro and animal models.

Pre-Clinical Studies	Treatment	Parameters Evaluated	Effect	References
Mouse glomerular mesangial cells (SV-40 MES 13)	Acetate, Butyrate, GPR43 agonist	Cell viability assays, detection of ROS, MDA and SOD, MCP-1, IL-1β and ICAM-1	Butyrate, Acetate, and GPR43 agonist reduced inflammatory markers	Huang et al., 2017 [132]
Renal tubular epithelial cells	Butyrate, Propionate, Acetate	NF-κB activation, NO production, ROS production	SCFAs reduced inflammation and hypoxia and also modulated immune response	Andrade-Olivera et al., 2015 [133]
Bone marrow dendritic cells		Molecules CD80 and CD40		
Antigen-presenting cells from RAGKO mice		Proliferation of CD8+ and CD4+ cells		
Male C57BL/6 mice with AKI		Apoptosis assessment, immunohistochemical analysis, mitochondrial DNA, DNA methylation, NF-κB levels, TLR-4, IL-6, IFN-γ, TNF-α, TGF-β1, MCP-1, IL-1β, GSS/GSSH ratio	Acetate reducing ROS, cytokines and chemokines; Low mRNA levels of TLR-4, lower activation of the NF-κB pathway, low levels of activated neutrophils and macrophages, a low frequency of infiltrating macrophages and a low frequency of activated DCs were observed. Acetate also increased the expression of GPR43 and inhibited the activity of HDACs	
Immortalised human renal proximal tubular epithelial cells (HK-2 cells)	Butyrate	TGF-β1 levels and expression	Butyrate reduces the basal generation of TGF-β1 through inhibition of the ERK/MAP kinase	Matsumoto et al., 2006 [137]
Wild-type and knockout C57BL/6 mice for GPR41, GPR43, or GPR109A receptors with diabetic nephropathy	Propionate, Butyrate	Assessment of serum creatinine, histological examination of renal tissues, evaluation of microbial composition, and SCFA levels, gene expression analysis of TLR-2, TLR-4, NLRP3, TNF-α, IL-6, IL-18, IL-1β, IL-4, IL-10, IFNγ, CXCL2, CCL2, CXCL10, iNOS, KIM1, MMP2, MMP9, TGFβ1, HDAC1-11, and GAPDH	Reduced inflammation and kidney injury, increased Bifidobacterium and Prevotella that increased faecal and serum SCFA concentrations	Liu et al., 2021 [140]
Mouse glomerular mesangial cells (SV-40 MES 13)	Acetate, Propionate, Butyrate	Cell viability assays, detection of ROS, MDA and SOD, Western blot analysis or ELISA for GPR43, β-arrestin-2, NF-κB, p65, MCP-1, IL-1β, I-κBα, GAPDH	Butyrate restored high glucose concentrations, oxidative stress, NF-κB signalling, and interaction between β -arrestin-2 and I-κB α-induced GPR43	Huang et al., 2020 [141]
Eight-week-old male C57BL/6 mice with type 2 diabetes induced by streptozotocin, diabetic nephropathy	Acetate, Propionate, Butyrate	FBG levels, ACR, FINS, BUN, SCr, serum cystatin C, TC, TG, LDL and LDL-C, renal glomerular histology, immunohistochemical staining for GPR43, β-arrestin-2, NF-κB, p65, MCP-1	Butyrate improved hyperglycemia, improved insulin resistance of T2D, prevented renal dysfunction in T2D, inhibited DT2-induced renal NF-κB activation and regulated GPR43- β -Arrestin2-signalling	
Renal tubular epithelial cells and podocytes were isolated from C57BL/6 mice	Acetate, Propionate, Butyrate	mRNA expression of IL-6, IFN-γ, TNF-α, CCL2, CXCL10	Butyrate and propionate significantly inhibited inflammation	Li et al., 2020 [142]
Wild-type and knockout C57BL/6 mice lacking genes for GPR43 or GPR109A with diabetic nephropathy	Fibre-rich diets, Sodium Acetate, Sodium Propionate, and Sodium Butyrate	Immunohistochemical analysis, histological analysis, real-time PCR for TLR-2, TLR-4, IL-6, IFN-γ, TNF-α, CCL2, CXCL10, TGF-β1, fibronectin, and GAPDH, bacterial DNA sequencing analysis, analysis of SCFA levels	Increased Prevotella and Bifidobacterium, increased SCFA, modulation of inflammation in renal tubular cells and podocytes under hyperglycemic conditions	
33 7-week-old male ICR with CKD adenine-induced	DF, GG, PHGG	Evaluation of TNF- α, MCP-1, IL-1β, IL-6, Tgfb1, Col1A1, Acta2, TLR-4 and Myd88 mRNA levels, expression levels of ZO-1, ZO-2, occludin, JAMA, claudin 3, claudin 4, and claudin 7, serum creatinine and urea analysis, immunohistochemistry analysis, microbiota composition analysis, SCFA levels, IgA of the mucosa and the mucus itself	Colonic barrier protection and reduced endotoxemia, restoration of tight junction protein expression and localisation, increased Bifidobacterium, increased propionic and butyric acid production correlated with a reduction in pro-inflammatory parameters	Hung et Suzuki, 2018 [145]
Seven-week-old male C57BL/6J mice with CKD adenine-induced	XOS	Col1A1, Cgtf, IL-6, TNF-α, Arg, Ym1, Defa, Pla2g2a, Reg3γ, caecal SCFAs, histological analysis of renal tissue	Reduced gene expression of markers observed in CKD, including lColA1 and Cgtf, IL-6 and the M2 macrophage marker, Defa5, reduced IS and pCS and increased SCFA-producing bacteria, and improved renal function	Yang et al., 2018 [144]
Male isogenic Balb/c mice and C57BL/6 with nephropathy	Diet of high amylose butyrate-releasing corn starch	Immunohistochemical analysis, DNA expression, analysis TGF-β1, Fsp1, ActaII, Col4α1, Mmp9, Timp1, urinary albumin analysis, monoclonal antibody generation for GPR109A and GPR43, analysis of SCFAs concentrations, measurement of pro-inflammatory cytokines, isolation and polarisation of bone marrow-derived macrophages, HDAC activity, DNA methylation	Butyrate attenuates inflammation and renal fibrosis through its receptors GPR109A, GPR43, and GPR41	Felizardo et al., 2019 [143]

Abbreviations: ROS, Reactive Oxygen Species; SOD, Super Oxide Dismutase; MDA, Malondialdehyde; IL, Interleukin; MCP-1, Monocyte Chemoattractant Protein-1; ICAM-1, Intracellular Adhesion Molecule-1; NF-κB, Nuclear Factor Kappa-light-chain-enhancer of Activated B cells; NO, Nitric Oxide; TLR, Toll-Like Receptor; TNF-α, Tumor Necrosis Factor alpha; *TGF-β*, Transforming Growth Factor beta; INF-γ, Interferon gamma; MMP, Matrix Metallopeptidase; KIM-1, Kidney Injury Molecule; HDAC, Histone Deacetylase; GAPDH, Glyceraldehyde 3-Phosphate Dehydrogenase; GPR, G-Protein-coupled Receptor; FBG, Fasting Blood Glucose; ACR, Random Urine Albumin-Creatinine ratios; FINS, Fasting Insulin Levels; BUN, Urea Nitrogen Levels; SCr, Serum Creatinine; TC, Total Cholesterol; TG, Triglycerides; LDL, Low-Density Lipoprotein; LDL-C, Low-Density Lipoprotein Cholesterol; CCL2, C-C Motif Chemokine Ligand 2; CXCL10, C-X-C motif chemokine ligand 10; SCFAs, Short-chain Fatty Acids; Fsp1, Fibroblast-Specific Protein 1; Col4α1, Kidney Collagen type IV alpha 1; ActaII, Actin alpha 2, Smooth Muscle; Col1A1, Kidney Collagen type I; IgA, Immunoglobulin A; ZO, JAMA; Defa5, Ileal Defensins alpha; Pla2g2a, Phospholipase A2; Reg3γ, Regenerating islet-Derived Protein 3 gamma; Cgtf, Connective tissue growth factor; Arg, Arginase; XOS; Timp1, Metallopeptidase Inhibitor 1; IS, Indoxyl Sulfate; pCS, pCresil Sulfate.

**Table 3:** Clinical studies report a related improvement in SCFA levels both in in vitro and animal models.

Clinical Studies	Treatment	Parameters Evaluated	Effect	References
20 patients with CKD and related complications	Sodium Propionate	Biochemical analyse on sera for hs-CRP, IL-2, IL-6, IL-10, IL-17a, TNF-α, INFγ, TGF-β, IL-10, MDA, and endotoxins/lipopolysaccharides	Sodium Propionate reduced inflammatory markers and improved anti-inflammatory parameters	Marzocco et al., 2018 [147]
43 patients with CKD	LPD	Analysis of bacterial populations and IS and pCS levels	LPD modulates gut dysbiosis and positively impacts the outcome of patients with CKD	Wu et al., 2020 [158]
Patients in HD with CKD	RS2	Evaluation of biochemical and clinical parameters, analysis of serum and plasma samples, genomic sequencing analysis	Mitigate inflammation and oxidative stress in hemodialysis patients by positively altering SCFA-producing bacteria	Kemp et al., 2021 [156]
214 patients with CKD and CVD	Addition of valerate	Evaluation of anthropometric, biochemical and clinical parameters, measurement of plasma levels of SCFAs	The addition of valerate to a model of hypertension, diabetes mellitus, and other complications significantly improved there conditions of patients	Jadoon et al., 2019 [154]
60 patients with grade 3B-4 CKD	FD, MD, VLPD	Anthropometri, clinical, and biochemical parameters obtained from stool and serum samples	Increased Lachnospiraceae, Ruminococcaceae, Prevotellaceae, Bifidobacteriaceae, Coprococcus, and Roseburia forming butyrate, increased anti-inflammatory potential, improved intestinal permeability and systolic blood pressure, reduced Enterobacteriaceae pathogens and circulating levels of IS, pCS, and D-Lactate	Di Iorio et al., 2018 [157]

Abbreviations: hs-CRP, high-sensitivity C-reactive protein; IL, Interleukin; INF-γ, Interferon gamma; MDA, Malondialdehyde; *TGF-β*, Transforming Growth Factor beta; TNF-α, Tumor Necrosis Factor alpha; IS, Indoxyl Sulfate; pCS, pCresil Sulfate; CVD, Cardiovascular disease; HD, Hemodialysis; CKD, Chronic Kidney Disease; LPD, Low-Protein Diet; SCFA, Short-chain Fatty Acids; FD, Free Diet; MD, Mediterranean Diet; VLPD, Very-Low Protein Diet.
